# Supplementary figures and images for: Infection and RNA-seq analysis of a zebrafish tlr2 mutant shows a broad function of this toll-like receptor in transcriptional and metabolic control and defense to Mycobacterium marinum infection
Source: BMC Genomics. 2019 Nov 20;20:878. doi: 10.1186/s12864-019-6265-1 (PMC6869251; doi:10.1186/s12864-019-6265-1)

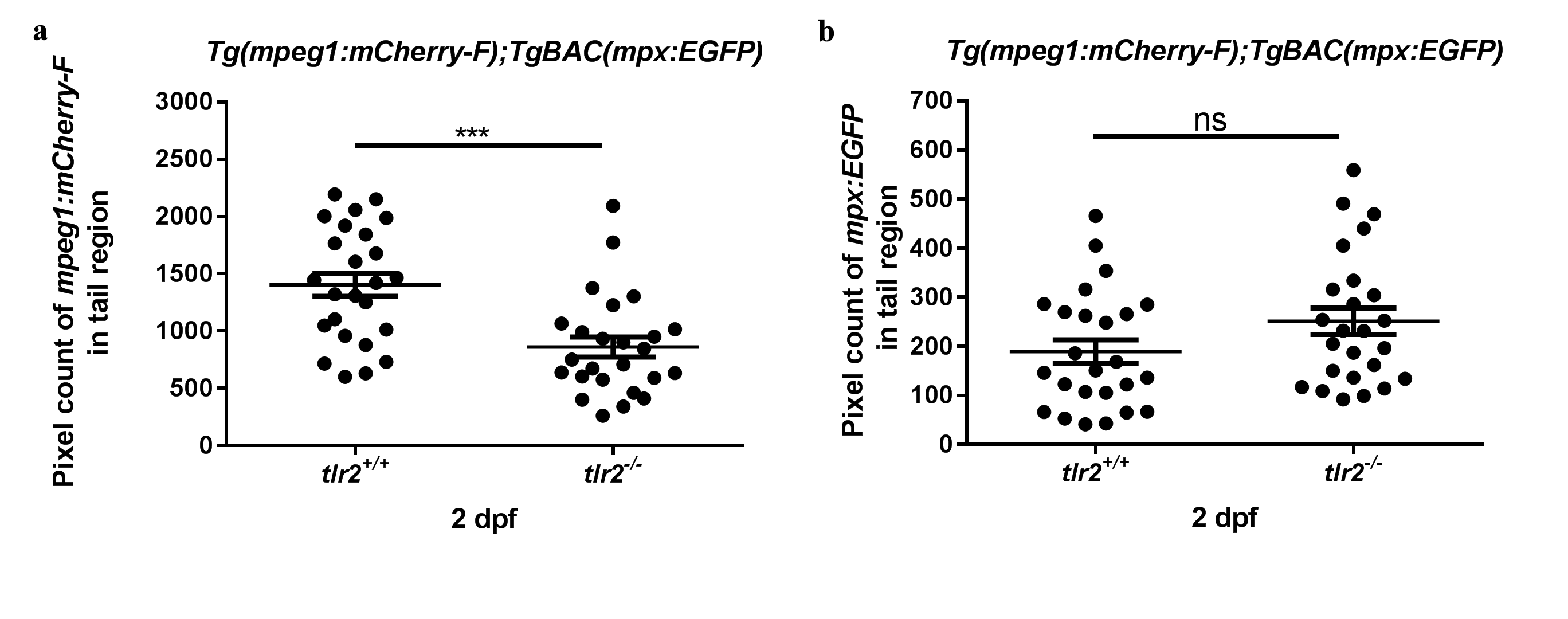

Supplement: Supplementary file 1 — Additional file 1: Figure S1. Pixel count analysis for double transgenic lines Tg (mpeg1:mCherry-F);TgBAC (mpx: EGFP) of 2 dpf tlr2+/+ and tlr2−/− embryos. a: mCherry reporter, b, EGFP reporter. [file 12864_2019_6265_MOESM1_ESM.tif]

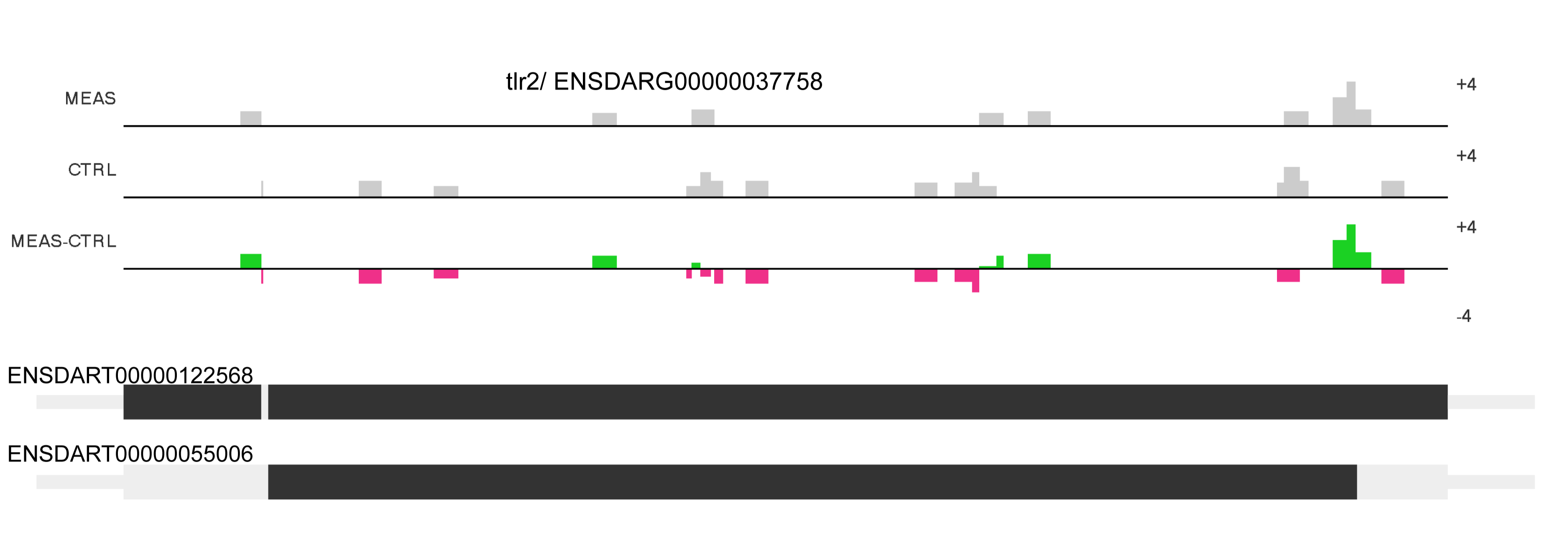

Supplement: Supplementary file 2 — Additional file 2: Figure S2. RNAseq read counts of tlr2 transcripts in heterozygotes (tlr2+/−) control versus tlr2 mutant (tlr2−/−) larvae. RNAseq data comparing reads mapped to tlr2 transcript (ENSDART0000012256). Heterozygotes and mutant reads are mapped to the entire length of the mutant transcript indicating that the mutant transcript is not subjected to nonsense mediated decay. Mutant data (MEAS) and heterozygotes (CTRL) data have been submitted to the NCBI gene expression Omnibus database, accession number is GSE102766. [file 12864_2019_6265_MOESM2_ESM.tif]

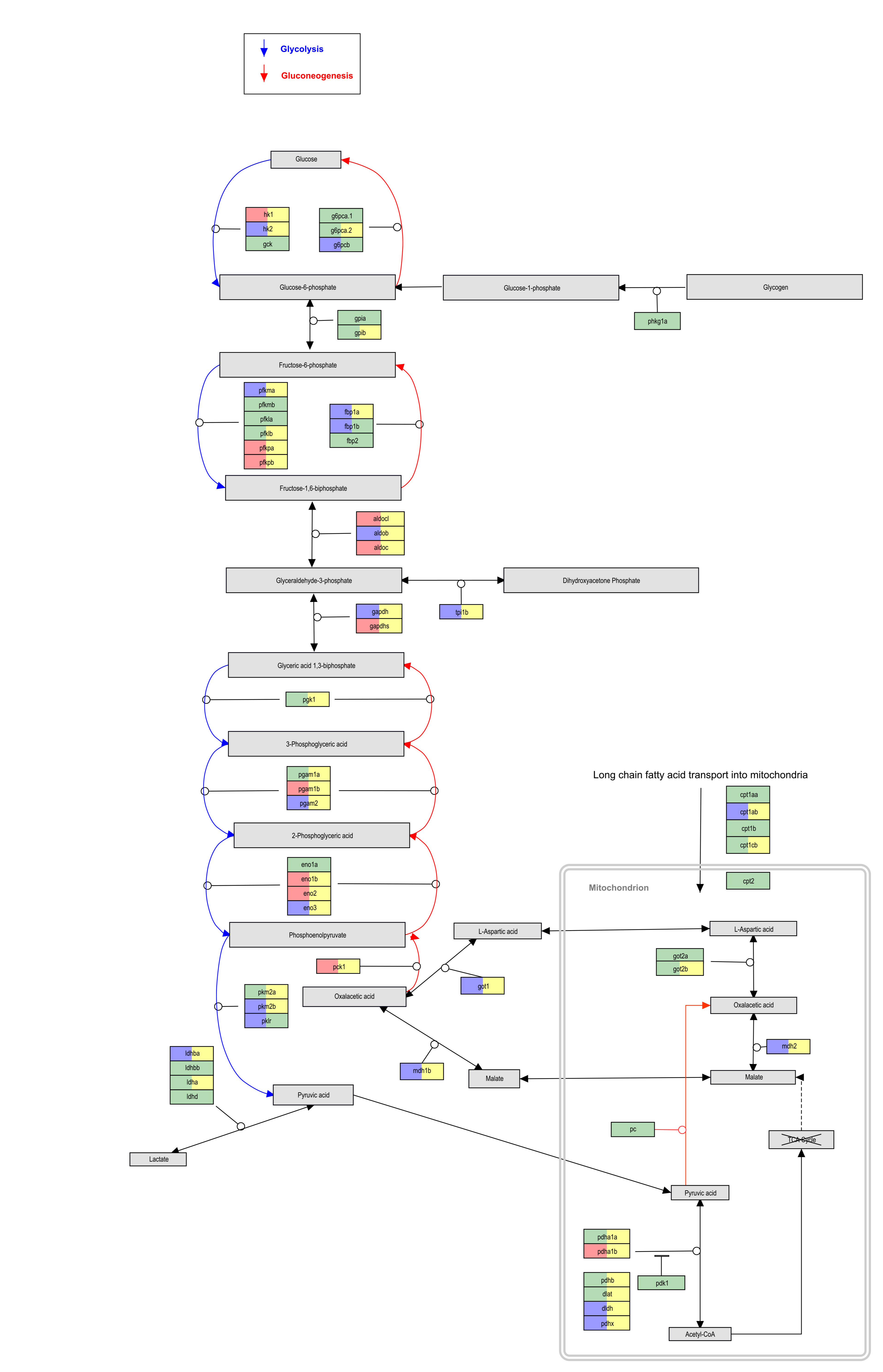

Supplement: Supplementary file 3 — Additional file 3: Figure S3. Analysis of differential expression of genes functioning in glycolysis and gluconeogenesis between uninfected tlr2+/− and tlr2−/−. The red boxes represent up regulated genes (FC > 2); blue boxes represent down regulated genes (FC < -2); yellow boxes represent the genes that are differentially expressed with a P value lower than 0.05; green represent not-significantly differentially expressed genes. [file 12864_2019_6265_MOESM3_ESM.tif]

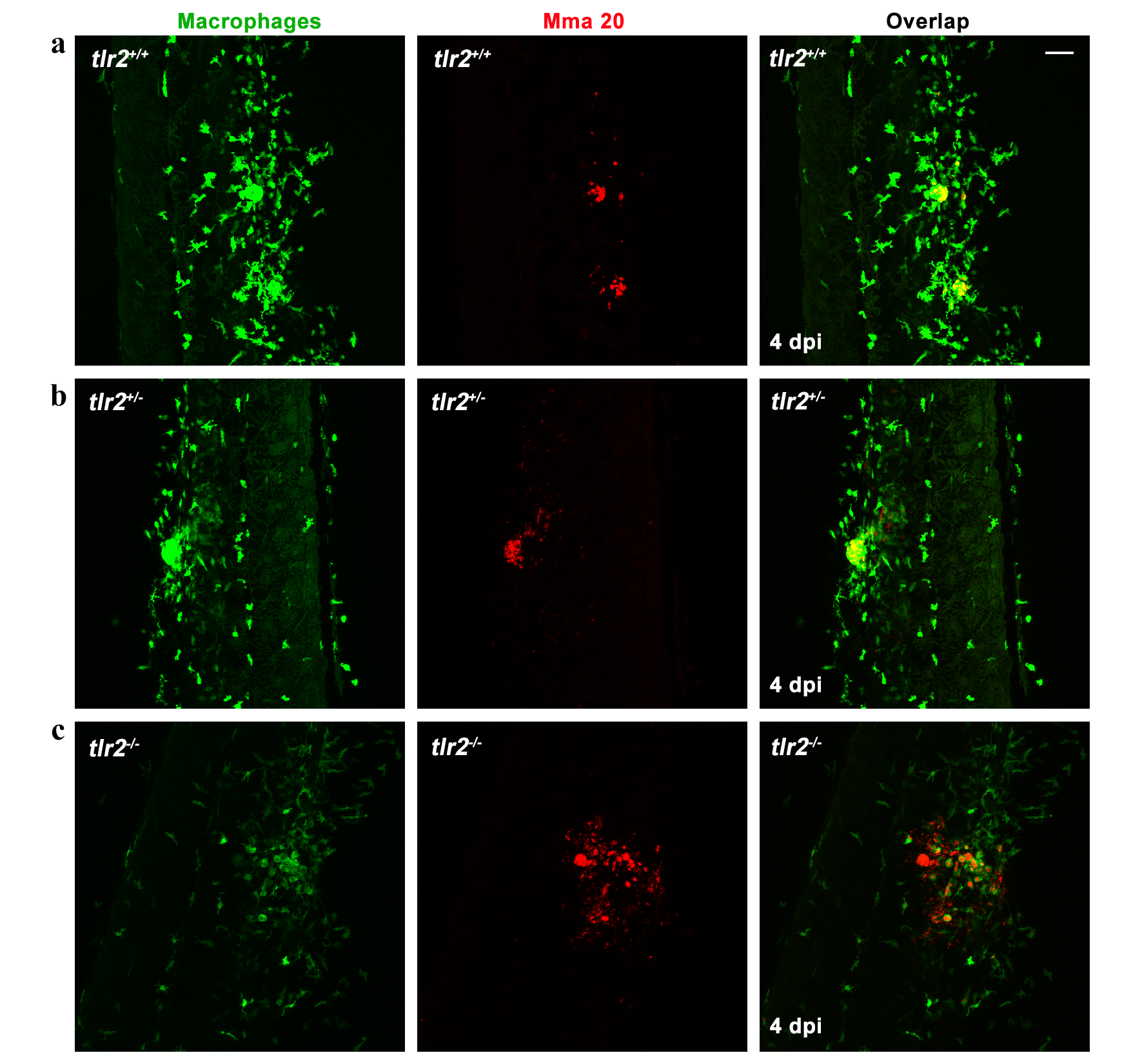

Supplement: Supplementary file 4 — Additional file 4: Figure S4. Representative images of the quantification of Mma20 infection phenotype in the tlr2 mutant, heterozygote and wild type siblings. One representative picture is shown for the entire sets of 38, 47 and 37 larvae were analysed for the tlr2 mutant, the heterozygote and the wild-type, respectively. Macrophages are in green and bacteria are in red. The scale bar represents 50 μm. [file 12864_2019_6265_MOESM4_ESM.tif]

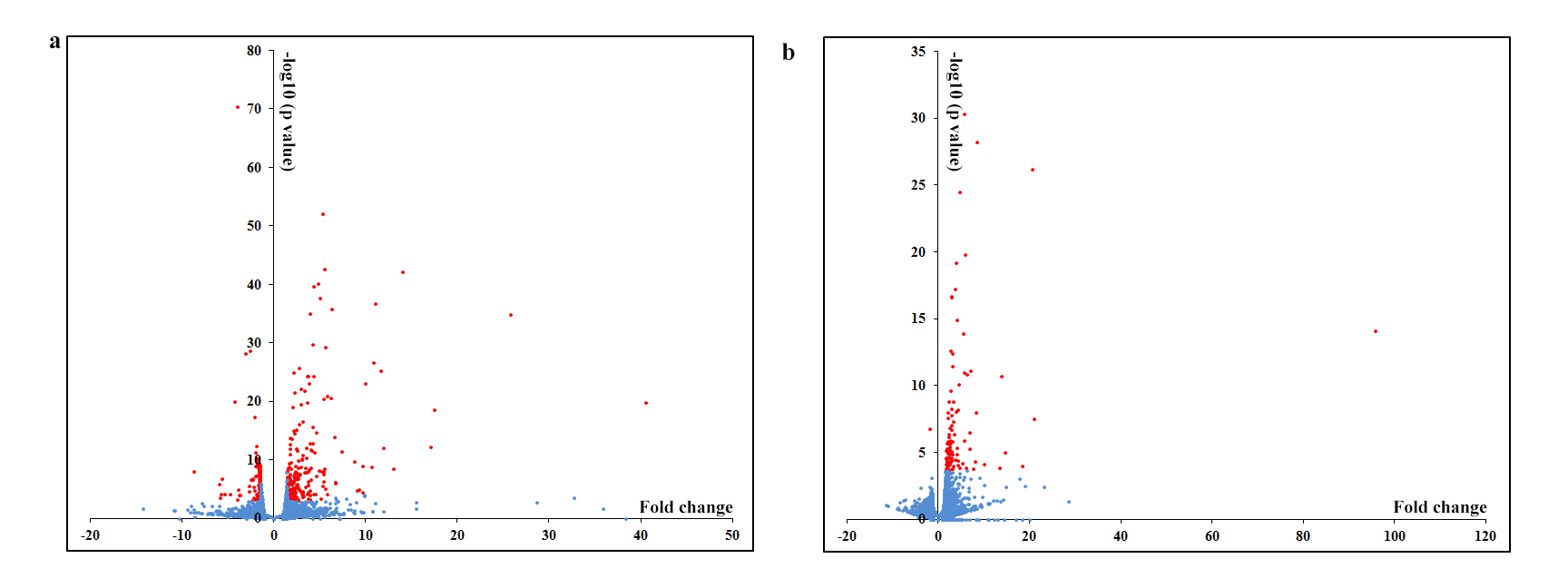

Supplement: Supplementary file 5 — Additional file 5: Figure S5. Volcano plots showing the significance cutoff applied to tlr2+/− infected with strain Mma20 versus control with PBS (a) and tlr2−/− infected with strain Mma20 versus control with PBS (b). In these volcano plots, the transcripts were considered significant (red) or non-significant (blue) by the conditions of |fold change| ≥ 1,45 and adjusted P value threshold ≤ 0,05. Fold changes for each transcript was plotted on the X-axis against -log10 transformed p-values on the Y-axis. [file 12864_2019_6265_MOESM5_ESM.tif]

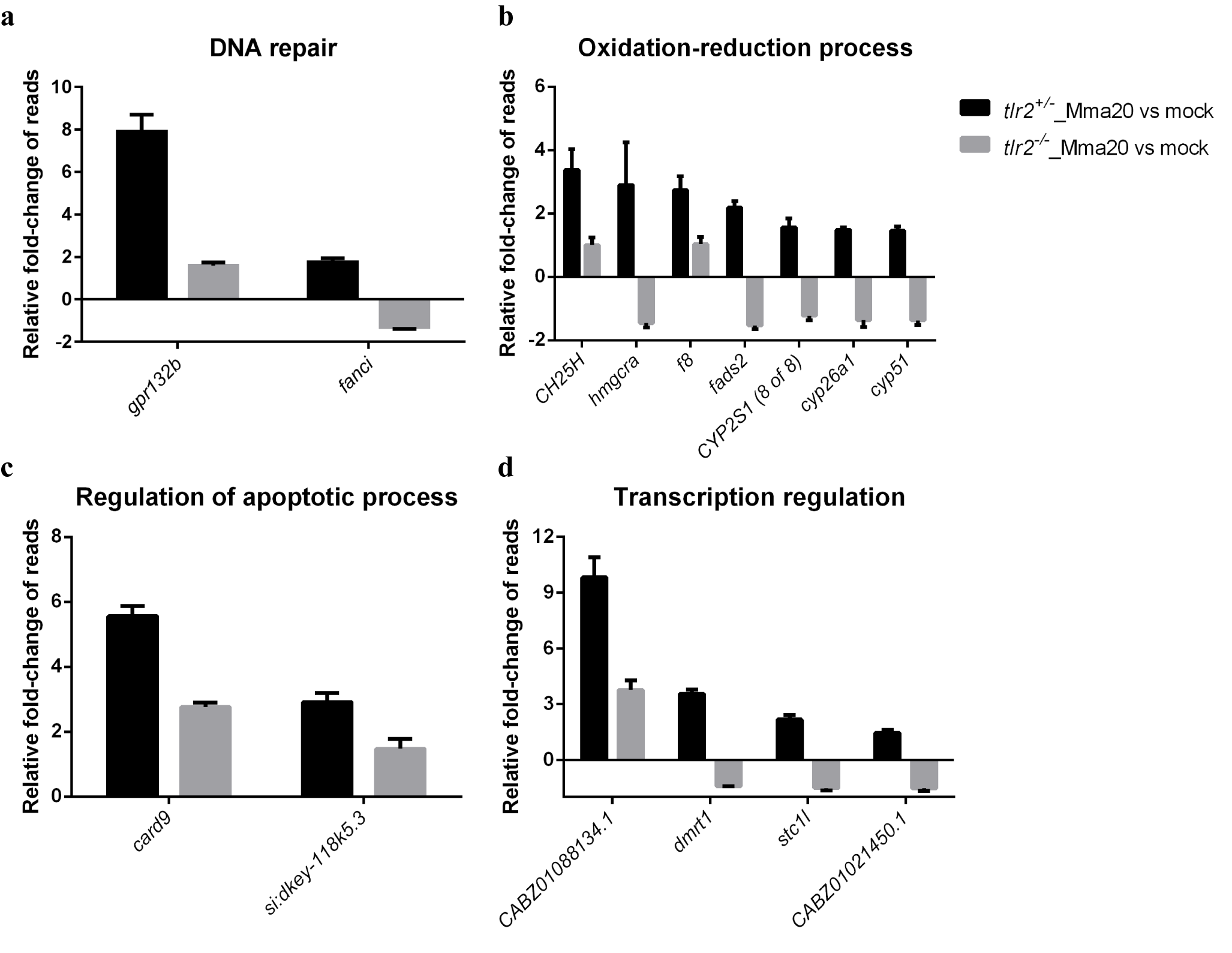

Supplement: Supplementary file 6 — Additional file 6: Figure S6. tlr2-dependent up regulation of genes with various GO terms. [file 12864_2019_6265_MOESM6_ESM.tif]

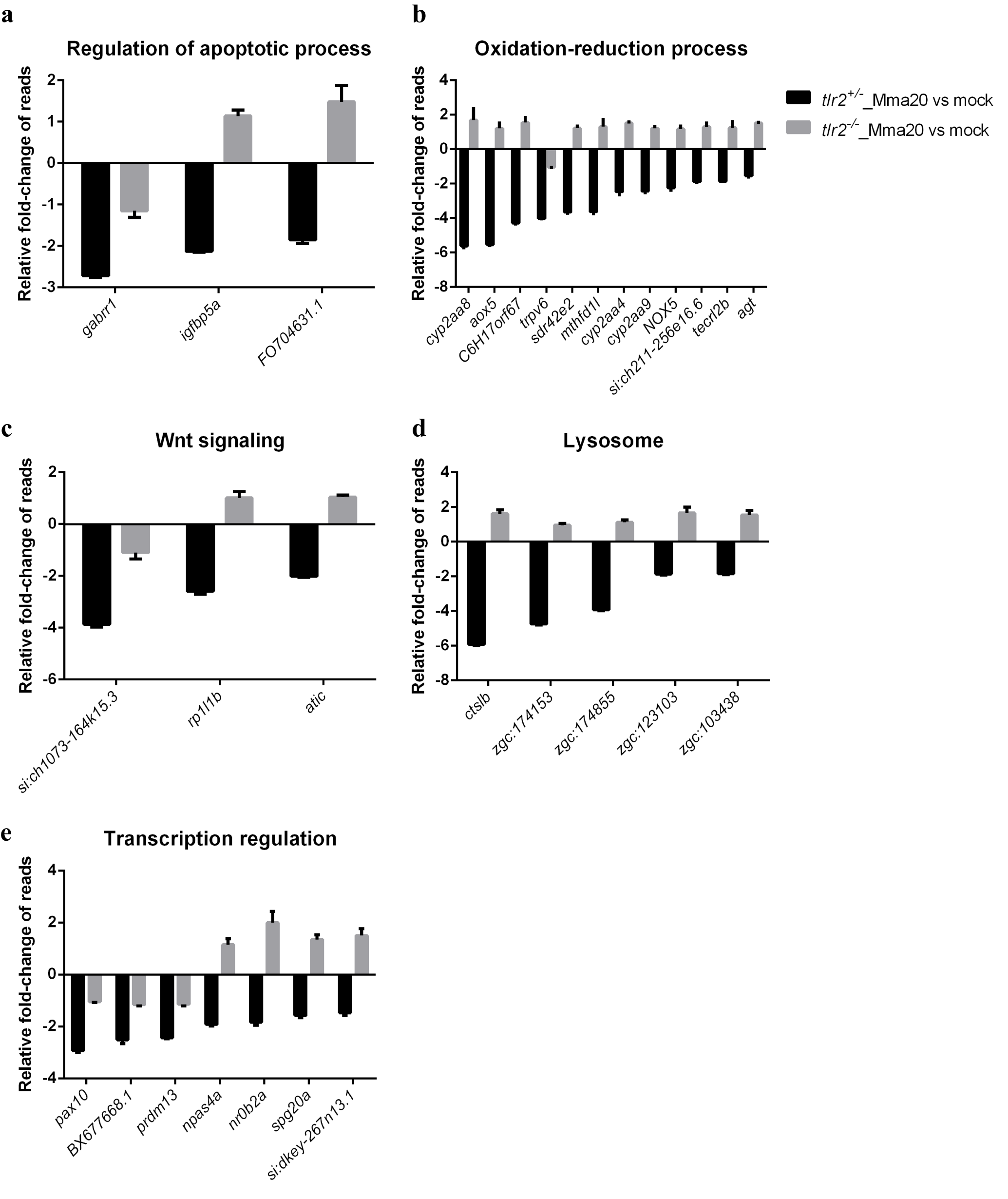

Supplement: Supplementary file 7 — Additional file 7: Figure S7. tlr2-dependent down regulation of genes with various GO terms. [file 12864_2019_6265_MOESM7_ESM.tif]

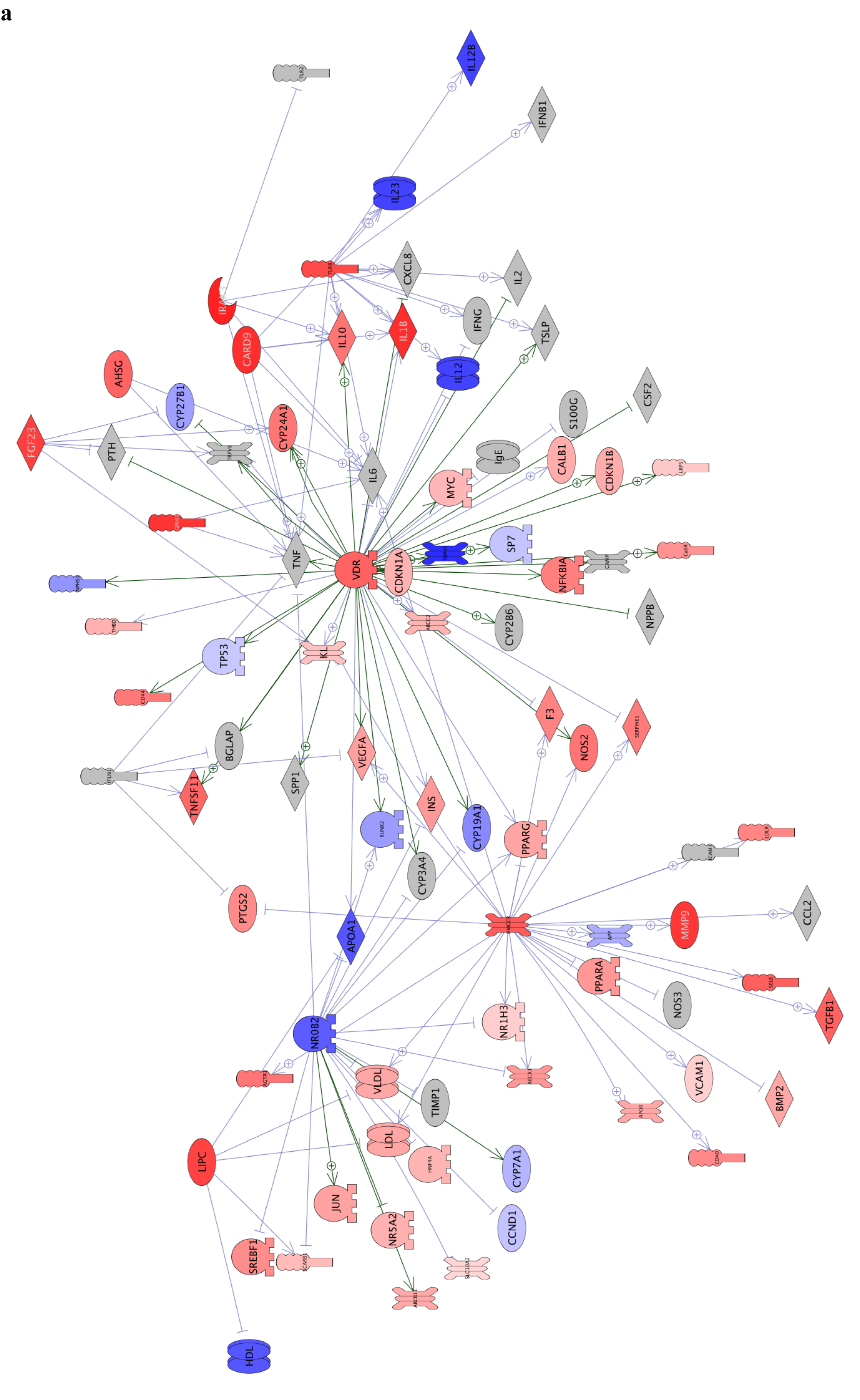

Supplement: Supplementary file 8 — Additional file 8: Figure S8. Sub-network enrichment analysis. Networks of common targets of the 97 up regulated genes (Fig. 6d) in tlr2+/− (a) and tlr2−/− (b) with Mma20 infection. Red represents up regulation, blue represents down regulation and grey represents genes for which no expression was detected. [file 12864_2019_6265_MOESM8_ESM.zip › Supplementary Fig 8a.tif]

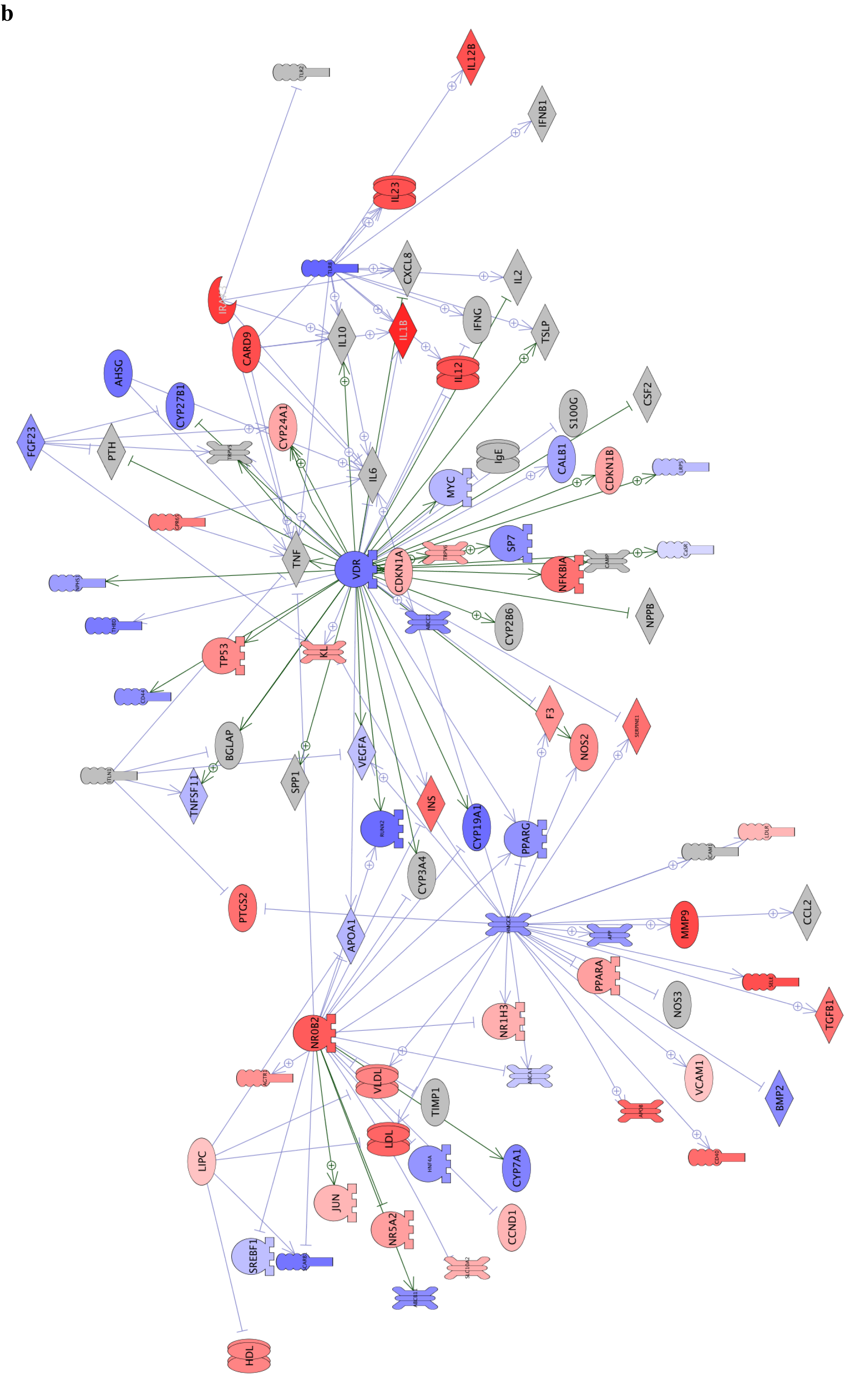

Supplement: Supplementary file 8 — Additional file 8: Figure S8. Sub-network enrichment analysis. Networks of common targets of the 97 up regulated genes (Fig. 6d) in tlr2+/− (a) and tlr2−/− (b) with Mma20 infection. Red represents up regulation, blue represents down regulation and grey represents genes for which no expression was detected. [file 12864_2019_6265_MOESM8_ESM.zip › Supplementary Fig 8b.tif]

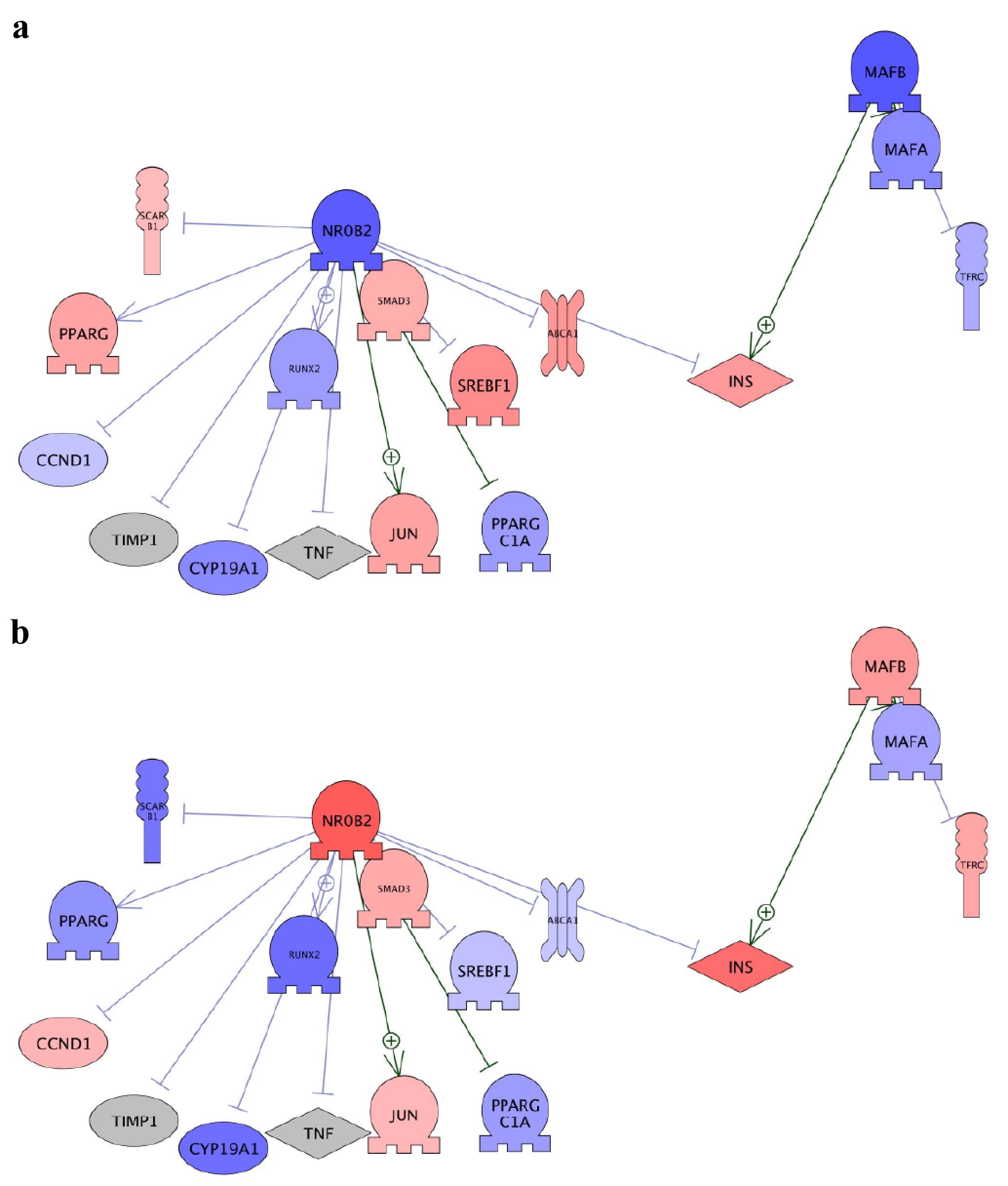

Supplement: Supplementary file 9 — Additional file 9: Figure S9. Sub-network enrichment analysis. The networks of common targets of the 92 down regulated genes (Fig. 6e) in tlr2+/− (a) and tlr2−/− (b) with Mma20 infection. Red represents up-regulation, blue represents down-regulation and grey represents genes for which no expression was detected. [file 12864_2019_6265_MOESM9_ESM.tif]

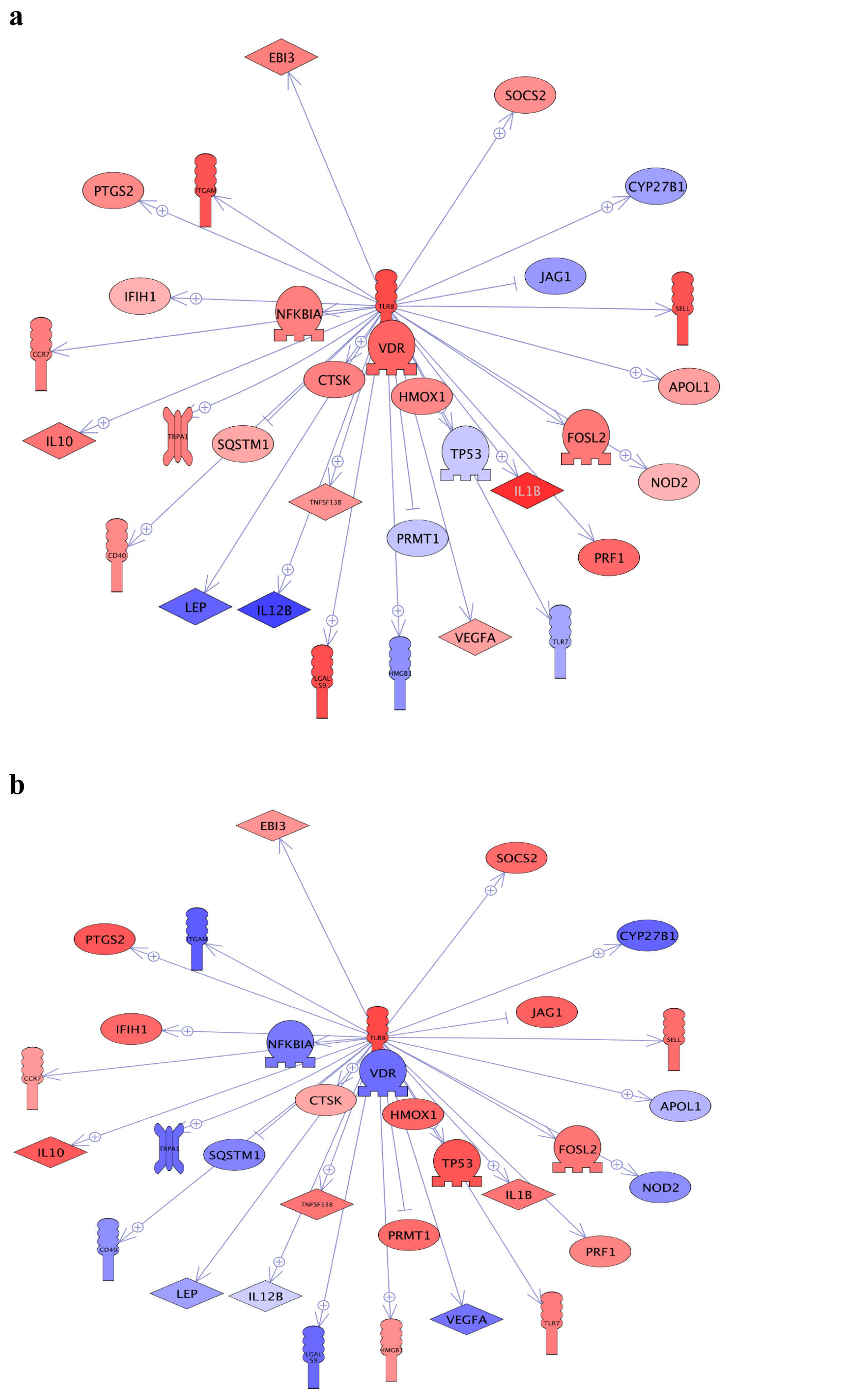

Supplement: Supplementary file 10 — Additional file 10: Figure S10. Sub-network enrichment analysis between zebrafish and human. The Tlr8 pathway in zebrafish (a) with Mm infection and human macrophages (b) with Mtb infection. Red represents up-regulation, blue represents down-regulation. [file 12864_2019_6265_MOESM10_ESM.tif]

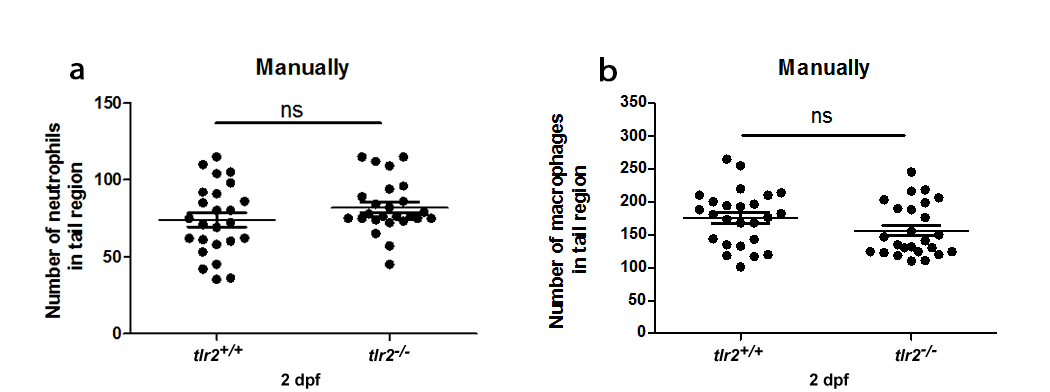

Supplement: Supplementary file 11 — Additional file 11: Figure S11. Manual counting analysis for Tg (mpeg1:mCherry-F);TgBAC (mpx: EGFP) of neutrophils (a) and macrophages (b) in 2 dpf tlr2+/+ and tlr2−/− embryos. [file 12864_2019_6265_MOESM11_ESM.tif]
